# Cutting Force Predication Based on Integration of Symmetric Fuzzy Number and Finite Element Method

**DOI:** 10.1155/2014/234150

**Published:** 2014-03-20

**Authors:** Zhanli Wang, Yanjuan Hu, Yao Wang, Chao Dong, Zaixiang Pang

**Affiliations:** ^1^School of Mechatronic Engineering, Changchun University of Technology, Changchun 130012, China; ^2^College of Mechanical Engineering, Beihua University, Jilin 132021, China

## Abstract

In the process of turning, pointing at the uncertain phenomenon of cutting which is caused by the disturbance of random factors, for determining the uncertain scope of cutting force, the integrated symmetric fuzzy number and the finite element method (FEM) are used in the prediction of cutting force. The method used symmetric fuzzy number to establish fuzzy function between cutting force and three factors and obtained the uncertain interval of cutting force by linear programming. At the same time, the change curve of cutting force with time was directly simulated by using thermal-mechanical coupling FEM; also the nonuniform stress field and temperature distribution of workpiece, tool, and chip under the action of thermal-mechanical coupling were simulated. The experimental result shows that the method is effective for the uncertain prediction of cutting force.

## 1. Introduction

The cutting force is an important physical parameter in the machine work; the predictive simulation study of the cutting force has already become one of the physical simulation fundamental studies of NC machining [1]. Nevertheless, the factors which can influence the cutting force in the actual machining process are very multitudinous and the relations between them are often varied and uncertain, so it is very difficult to establish a perfect prediction model [2, 3]. At present, the great majority of prediction models (such as empirical formula and neural network) are at the cursory level of the qualitative analysis. However, the cutting force from the actual measurement is an uncertain value which is fluctuated in certain range instead of a certain value, so that the uncertainty study to the cutting force needs to be solved imminently.

Currently, the common methods for estimating the uncertainty of system are probability distribution method, fuzzy estimation method, least square method, and so on; for example, Axinte proposed the TLSRM model based on the least square regression method and the theory of error compensation, which provided a certain value for the prediction result of uncertainty [4]. However, the cutting force from the actual measurement is an uncertain value which is fluctuated in certain range, consequently, in order to truly evaluate the uncertainty of the cutting force and establish a prediction model of cutting force with high prediction accuracy, the method that uses the symmetric fuzzy number to predict the cutting force is adopted. This method can establish a fuzzy forecasting model called GFPM, which brings a scope instead of a certain value as its prediction result for the uncertainty of cutting force. From the view of engineering practice, the cutting force prediction that is in form of scope has more realistic meaning than in the form of certain value. At the same time, using the method which combined the thermomechanical coupled finite element for the cutting force prediction of the actual machining process, both the cutting force can be measured and the state variable which is difficult to be directly measured or cannot be achieved by experiment can be got, for example, the distribution of stress and temperature field, and so forth. Comparing the finite element method with measured value, the analysis result shows the accurate simulation can be fleetly achieved by finite element method. The experimental proof that the cutting force prediction integrated the symmetric fuzzy number and thermomechanical coupled finite element method into it can give a prediction scope of cutting force by estimating the uncertainty of cutting force as well as quantificationally predict the cutting force.

## 2. The Cutting Force Fuzzy Prediction for the Symmetric Fuzzy Number

In the practical considerations, the gather of the important influencing factor for dependent variable *y* has already be ensured as *X*_*p*_ = {*x*_*p*1_, *x*_*p*2_,…, *x*_*pn*_}, so that *Y*(*X*_*p*_) is the ambiguity function of *X*_*p*_ [5]; that is

(1) Y(Xp)=A0+A1xp1+A2xp2⋯Anxpn,p=1,2,3,…,m.

In the type, *Y*(*X*_*p*_) is the symmetric fuzzy number, which shows the output of fuzzy linear model; *m* is the number of the experimental classes; *A*_*i*_  (*i* = 0,1,…, *n*)  is the fuzzy number of symmetric triangular, which shows *A*_*i*_  (*a*_*i*_, *c*_*i*_), and its membership function is

(2) $$\begin{array}{c} \mu_{A_{i}}\left(x\right)=\left\{\begin{array}{cc} 1-\frac{\left|x-a_{i}\right|}{c_{i}} & \left|x-a_{i}\right|\leq c_{i}\\ 0 & \text{else} \end{array}\right\}. \end{array}$$

In the type, *A*_*i*_ means centering the membership function around *a*_*i*_; according to the referenced function *L*(*x*) = max⁡{0, 1 − |*x*|}, dispersing the distance of *C*_*i*_ symmetrically, *a*_*i*_ is called of central value of *A*_*i*_, and *C*_*i*_ is the fuzzy magnitude of *A*_*i*_.

## 3. The Simulation of Cutting Force Based on Thermomechanical Coupled Finite Element Method

The simulation of cutting force, which is based on thermomechanical coupled finite element method, contains the Geometric modeling, material constitutive model, grid technology, chip separation criteria, the contact friction characteristics, and conduction equation of cutting heat.

### 3.1. Geometric Modeling

**Figure 1:**
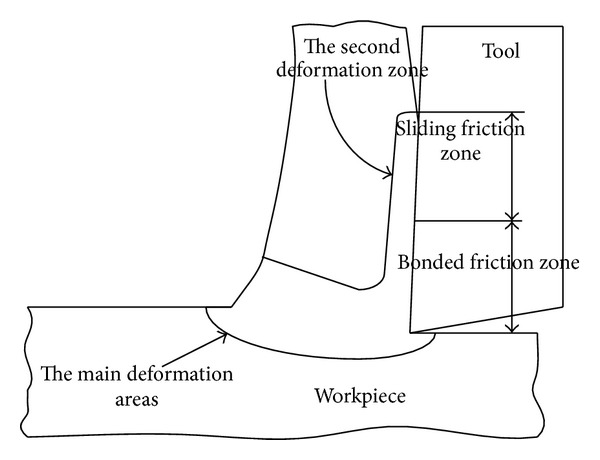
Two-dimensional model of cutting plan.

In actual turning process, the main cutting edge will be involved in cutting as well as the end cutting edge, synthetically considering, if both the main cutting edge and end cutting edge are involved in the turning process in the finite element simulation, in that way, there will bring an interference at the join of main cutting edge and end cutting edge by the metal layer, so that the simulation cannot go on wheels. In order to solve this problem, the 2D orthogonal cutting model is adopted (shown in Figure 1). This model assumes the cutting edge perpendicular to the cutting speed in the whole cutting process, and the main straight cutting edge is involved in the cutting alone, the end cutting edge does not partake in the cutting process, the deformation of metal cutting is approximately thought of as 2D deformation, and the strain and stress in the deformed area will not change along the *z*-axis.

### 3.2. Constitutive Model of Material

In the cutting process, the metallic material will have plastoelastic deformation under the mixing effects of high pressure, high temperature, high stress, and so forth, and therefore, it is needed that effect of every factor is considered when simulating. In order to describe the physical characteristics of material in process-cycle, the constitutive model called Johnson-Cook is adopted, which is used under the conditions of metal large deformation, high strain rate effect, and high temperature. The equation structure is simple and has wide range of applications, and it is applied in the station of the wide range of strain rates [6]. The representation of the constitutive model called Johnson-Cook is shown as follows:

(3) $$\begin{array}{c} \overset{-}{\sigma }=\left[A+B{\left(\dot{\overset{-}{\varepsilon }}\right)}^{n}\right]\left[1+C\ln\left(\frac{\dot{\overset{-}{\varepsilon }}}{{\dot{\overset{-}{\varepsilon }}}_{0}\left(S^{-1}\right)}\right)\right]\left[1-{\hat{\theta }}^{m}\right],\\ \hat{\theta }=\left\{\begin{array}{cc} 0 & \theta <\theta_{t}\\ \frac{T-T_{0}}{T_{\text{melt}}-T_{0}} & \theta_{t}\leq \theta \leq \theta_{m}\\ & \theta >\theta_{m} \end{array}\right\}. \end{array}$$

In the type, $\overset{-}{\sigma }$ is the equivalent effective stress; $\overset{-}{\varepsilon }$ is the equivalent plastic strain; $\dot{\overset{-}{\varepsilon }}$ is the plastic strain rate; ${\dot{\overset{-}{\varepsilon }}}_{0}$ is the referenced strain rate (1.0 s^−1^); yield strength *T*_0_ is the room temperature; *T*_melt_ is the fusion temperature; *A* is the (MPa) of material, *B* is the hardening modulus (MPa) of material; *n* is the strengthening factor of strain; *C* is the specific strength of strain rate, *m* is the coefficient of thermal softening. *C*, *n*, and *m* are characterization factors of material, and they can be achieved by material experiment or cutting experiment. Pointing at the finite element simulation in the cutting process of the AISI-1045 steel, the natural parameter of Johnson-Cook model for AISI1045 steel is established, which is concretely shown as Table 1.

### 3.3. Technology of Grid Division

The finite element simulation in the cutting process of metal is a typical local deformation process; the geometry and size of workpieces are time-variant and will be aggravated by deformation, so that the grids will produce twist and deformation, when they reach a certain limit; then they will deduce the analysis accuracy and shorten the stable step, and even the simulation can be destroyed when the twist and deformation is serious. For avoiding the germination of this problem, the technology of any Lagrange-Euler adaptive grid division is adopted, which has combined the advantages of Lagrange method and Euler method, so that the form of grids can self-adaptively change along with the workpieces deformation; the distortion of grid in the simulation process can be avoided consequently.

### 3.4. Cutting Separation Rule

In the actual machining process, the workpiece material continually separate from the rough carving, which will become smear metal. It needs to take the situation of smear metal into consideration when the simulation of the actual cutting process is established. The Johnson-Cook fault equation simulates the separation of smear and workpiece by a dynamic failure model (i.e., is when the damage parameter *ω* = 1, the unit material loses effectiveness); this method is failure criteria that synchronously take the strain, strain rate, temperature, and stress into consideration, which is adaptive for the analysis of the metal deformation with high strain rate, and its advantage is the combine with experiment, so that it can get the higher reliability [8]. Its damage parameter is defined as follows:

(4) $$\begin{array}{c} \omega =\sum \left(\frac{\Delta {\overset{-}{\varepsilon }}^{\text{pl}}}{{\overset{-}{\varepsilon }}_{f}^{\text{pl}}}\right),\\ {\overset{-}{\varepsilon }}_{f}^{\text{pl}}=\left[d_{1}+d_{2}\exp\left(d_{3}\frac{\sigma_{p}}{\sigma_{q}}\right)\right]\left[1+d_{4}\ln\left(\frac{{\dot{\overset{-}{\varepsilon }}}^{\text{pl}}}{{\dot{\varepsilon }}_{0}}\right)\right]\left(1+d_{5}\hat{\theta }\right). \end{array}$$

In (4), ${\overset{-}{\varepsilon }}_{f}^{\text{pl}}$ is the failure strain; ${\dot{\overset{-}{\varepsilon }}}^{\text{pl}}/{\dot{\varepsilon }}_{0}$ is the dimension of plasticity strain rate; *p*/*q* is the dimension of the rate of compression stress and deviatoric stress; *σ*_*p*_/*σ*_*q*_ is the dimensionless bias stress ratio; $\hat{\theta }$ is the dimensionless temperature;* d*_*1*_~*d*_*5*_ is the failure parameter under the condition of altered temperature, which can be achieved by the tensile and torsional experiment; ${\dot{\varepsilon }}_{0}$ is the reference strain rate.

Table 2 is the description of the Johnson-Cook specified failure parameters of AISI-1045 steel.

### 3.5. Characteristics of Contact Friction

As the result of the friction extrusion between the rake face, smear metal, flank of the cutter, and the finished surface brings big influence to the wear of cutters and the working accuracy of workpieces, the established finite element model can truly reflect the highly nonlinear contacted situation between the rake face and workpiece. The friction model proposed by Zorev [9] shows that there are two different contacted states; they are sliding zone and bonded zone; the shearing stress of every point in bonded zone is basically identical; the friction stress in the sliding zone is decreased along the anterior angle of the cutter, which has met the friction rule called Runcu. That is

(5) $$\begin{array}{c} \tau_{f}=\left\{\begin{array}{cc} \mu \sigma_{n} & \mu \sigma_{n}<\left(\text{sliding}\;\;\text{friction}\;\;\text{zone}\right)\\ \tau_{S} & \mu \sigma_{n}\geq \left(\text{bonded}\;\;\text{friction}\;\;\text{zone}\right) \end{array}\right\}, \end{array}$$

where *τ*_*f*_ is the friction stress of the smear metal interface, *μ* is the friction factor, *σ*_*n*_ is the direct stress of the smear metal interface, and *τ*_*S*_ is the sheared flow stress of the cutting material.

### 3.6. Equation of Heat Conduction

In the temperature field, numerous influencing factors and boundary conditions in the deformed area of the metal are comparatively complicated. It is difficult to get the solution by the traditional analytic method and numerical method, but the heat source method has its originality; especially the scope of heat conduction is immeasurably extensive, but the heat source is included in the infinitesimal microvolume; the heat source can get the most simple solution, and its computing result is confoundedly identical with the actual result [10]. So the temperature field of the deformed area can be analysed by the heat source method. The partial differential equation of heat conduction is

(6) $$\begin{array}{c} \lambda \frac{\partial^{2}\theta }{{\partial x}^{2}}+\lambda \frac{\partial^{2}\theta }{{\partial y}^{2}}-\rho C_{p}\left(\mu_{x}\frac{\partial \theta }{\partial x}+\mu_{y}\frac{\partial \theta }{\partial y}\right)+\dot{Q}=0,\\ \dot{Q}=\frac{W_{h}\dot{\overset{-}{\varepsilon }}\overset{-}{\sigma }}{J}. \end{array}$$

In (6), *λ* is the heat conduced rate, *C* is the specific heat, *ρ* is the density of the material, $\dot{Q}$ is the rate of the heat generation, *W*_*h*_ is the rate of the inversion which is plastic deformation to heat, $\overset{-}{\sigma }$ is the equivalent effective stress, and $\dot{\overset{-}{\varepsilon }}$ is the accelerant rate of equivalent effective stress.

## 4. Modeling Verification

**Figure 2:**
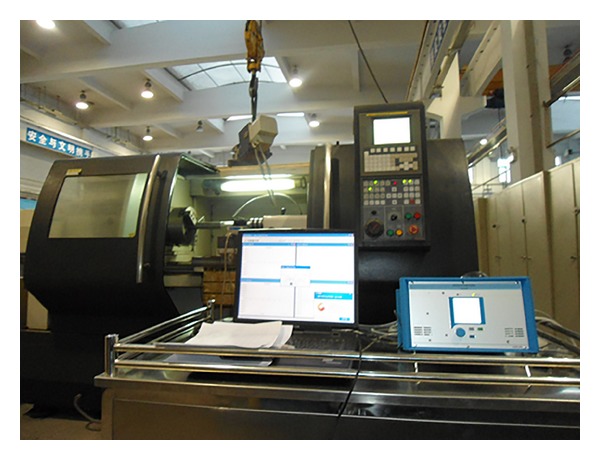
Experimental equipment.

Making the cutting experiment on the CAK5085dj lathe, the used dynamometer is KISTLER Type 9257B (the dynamometer rage is defined as 0~5 KN), the cutter material YT15, anterior angle is 15°, relief angle is 8°, and tool cutting edge inclination angle is 0°. The material of workpieces is 45^#^ steel and diameter of work is 45 mm (the equipment for the experiment is shown in Figure 2).

Table 3 shows the value of the cutting force measurement of AISI-1045 steel.

### 4.1. Prediction for Cutting Force of the Symmetric Fuzzy Number

There are a comparably large number of factors that influence the cutting; thereinto, the cutting speed *V*, back engagement of the cutting edge *a*_*p*_, and the load *f* have larger effect. So the fuzzy function between cutting force and the three elements according to (1) and its establishing process is as follows.

According the experimental equation of cutting force,

(7) $$\begin{array}{c} F_{Z}=C_{F_{Z}}{\left(a_{p}\right)}^{\alpha }{\left(f\right)}^{\beta }{\left(v\right)}^{\gamma }K_{F_{Z}}. \end{array}$$

Taking the logarithm for the both sides of (7)

(8) $$\begin{array}{c} \ln\;F_{Z}=\ln C_{F_{Z}}+\ln K_{F_{Z}}+\alpha \ln a_{p}+\beta \ln f+\gamma \ln v, \end{array}$$

assume *Y* = ln⁡*F*_*Z*_; *b*_0_ = ln⁡*C*_*F*_ + ln⁡*K*_*F*_; *X*_1_ = ln⁡*a*_*p*_; *X*_2_ = ln⁡*f*; *X*_3_ = ln⁡*v*; *b*_1_ = *α*; *b*_2_ = *β*; *b*_3_ = *γ* brought into (7), the clear form for linear model, which is

(9) $$\begin{array}{c} Y=b_{0}+b_{1}X_{1}+b_{2}X_{2}+b_{3}X_{3}. \end{array}$$

The fuzzy function for the defined equation (9) is

(10) Y(Xp)=A0+A1Xp1+A2Xp2+A3Xp3p=1,2,3,…,m,Y(Xp)=[ln⁡(FZ)]p=[C(Xp),W(Xp)],Ai=[Ci,Wi], i=0,1,2,3,C(Xp)=C0+C1Xp1+C2Xp2+C3Xp3,W(Xp)=W0+W1Xp1+W2Xp2+W3Xp3.

In the equation, *Y*(*X*_*p*_) is the fuzzy prediction value, *X*_*p*1_ = [ln⁡(*a*_*p*_)]_*p*_, *X*_*p*2_ = [ln⁡(*f*)]_*p*_ (*p* is defined as the experimental code, *p* = 1,2, 3,…, 10); *A*_*i*_ is fuzzy coefficient; *C*_*i*_ is the symmetric fuzzy number; *C*_*i*_ is the center of the symmetric fuzzy number; *W*_*i*_ is the breadth of the symmetric fuzzy number.

In order to make the fuzzy prediction force be included in the scope of (10), and minimizing the sum of the width of symmetric fuzzy number *W*_*i*_, the following linear planning should be satisfied.

The planning target is

(11) obj.min⁡ ∑W(Xp)St. Yp≤C(Xp)+L(h)W(Xp)Yp≥C(Xp)−L(h)W(Xp)L(h)=1−h0≤h<1, W≥0, C≥0.

Choosing the degree of membership *h* = 0.5 according to (11) and datasheet 1, the concrete model under the condition of (10) can be achieved by using the software of Matlab,

(12) Y(Xp)=[ln⁡FZ]p=A0+A1[ln⁡ap]p+A2[ln⁡f]p +A3[ln⁡v]p.

In equation (12),

(13)  A0=[0.000000,0.000000], A1=[0.2738294,0.000000], A2=[0.7705319,0.9722303E-01], A3=[0.3975155,0.000000].

Table 4 lists the prediction result of cutting force under the condition of the given cutting force. Based on the listed data, we can get that the actual cutting force is symmetrically included in the scope of fuzzy prediction. The experiment showed that the satisfied result of the degree of membership *h* in (11) can be achieved in the scope of [0.2, 0.7].

### 4.2. Simulation Analysis Based on the Thermomechanical Coupled Finite Element Method

**Figure 3:**
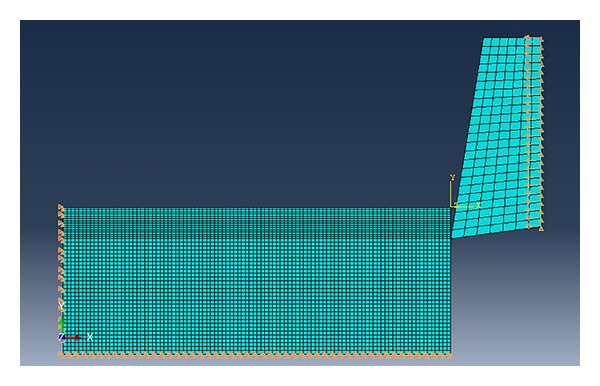
Finite element simulation model by ABAQUS.

Establish the 2D orthogonal cutting model, set the size of the workpiece as 20 mm × 8.5 mm, and set the initial temperature of workpiece and cutter as 20°C. The cutting speed is defined as 254 m/min and the direction of the speed is along the negative direction of *x*-axis, the cutting depth ap is equal to 0.75 mm, fixed to the panning and turning free degree on the direction of the *X*, *Y*, *Z* of the bottom margin and left of the workpiece (as shown in Figure 3).

#### 4.2.1. Stress Analysis

Normally, the forming process of the banding cutting can be classified in three stages, such as cut-over, taking shape, and steady forming.

**Figure 4:**
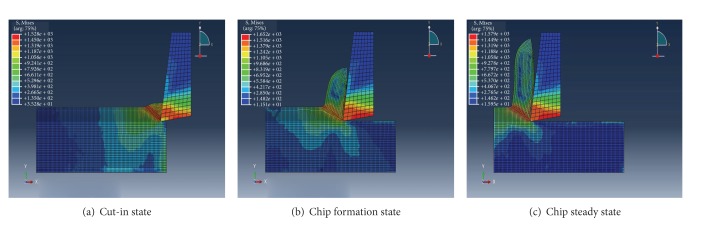
The stress distribution of cutting speed of 254 m/min.

Figure 4 shows the distribution of the equivalent stress of the workpiece and cutter in the three stages. The stress nephogram during cutting workpiece is equivalent stress expanding, with the pitching-in of the cutter; the cutter generally formed the largest equivalent stress zone in the first deformed area. After that, as the result of the character of the thermal softening, even though the deformation is aggravating constantly, the equivalent stress still has certain decline, which was shown by the instability of the material and the cutting process; at the same time, the value of the stress in the largest equivalent stress zone has little change (as shown in Figures 4(b) and 4(c)); the result of the simulation meets the yield criteria of Von Mises. Namely, when the material step is into the condition of plasticity, the equivalent stress is likely to remain unchanged. The equivalent stress nephogram of the cutter shows that the equivalent stress of the flank is larger than rake face, and the largest equivalent stress is produced at the knifepoint and the point closed to the knifepoint of the rank face; the extrusion and friction of the rank face are more serious. This can illustrate that the rank face is easier to be abraded in the actual cutting process.

#### 4.2.2. Temperature Analysis

**Figure 5:**
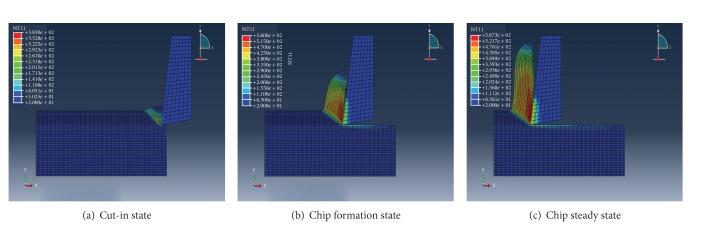
The temperature distribution of cutting speed of 254 m/min.

It is shown in the temperature field nephogram that the majority of the heat in the cutting process is carried off by the smear metal, and the small part is introduced in cutter. From the initial cutting to stable cutting time, the distribution of the temperature field has experienced four stages; initial phase (it is shown in Figure 5(a)), cutting heat is produced in the first deformation zone, and at smear metal of the point closed to the flank, there appeared the concentrated area of the temperature; this is because that, at the initial stage, the cutter mainly overcomes the higher cutting heat which is produced by applying work through plastic deformation in the first deformation zone; the formation stage of the smear metal (as shown in Figure 5(b)), the intensive zone of the cutting heat begins transform to the second deformation zone; at this time the temperature summit is at the point that is 2~3 mm away from knifepoint rather than knifepoint. This is because there was serious friction between the flank and smear metal; the friction heat makes the cutter contact increase the heat at the surface. With carrying on of the cutting process, the smear metal is formed in one stage (as shown in Figure 5(b)) because of the friction heat between the processed surface and rank face, the concentrated zone of cutting heat extends to the third deformation zone; at last, the smear metal forming comes into stable status (as shown in Figure 5(c)), the cutting heat of the second deformation zone and the third deformation zone is generally spread up along the flank and rightward along the rank face. This is because the cutting speed is too fast to make the cutting heat between the smear metal and the flank or the cutting heat between the processed surface and the flank face spread, resulting in residues on the smear metal and the processed surface.

#### 4.2.3. Simulation of Cutting Force

**Figure 6:**
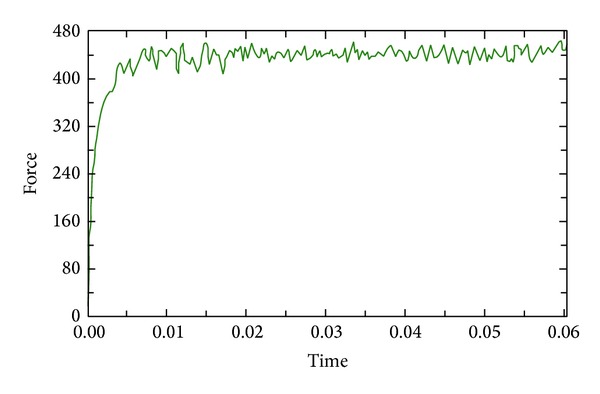
The main cutting force change curves when cutting speed is 254 m/min.

The simulation curve of the cutting force is shown in Figure 6, whereby the cutting force is sharply ascendant; while the cutter just cuts in the workpiece, the main cutting force tends to be stable generally over the constantly driving time, and the main cutting force tends to be stable at the cutting time of 0.0811 seconds. The value of the main cutting fluctuates from 400 N~480 N; this fluctuation is caused by the factor of contact, crispation, separation, or break, and so forth between smear metal and rake face.

#### 4.2.4. Experimental Verification

To verify the validity of the coupled thermomechanical finite element simulation results, the cutting experiment is carried out on the lathe named CAK5058dj. The turning condition is set as the cutting speed *V* at 254 m/min, diameter of work is defined as 45 mm, the material of work is the steel of AISI-1045 (the experimental conditions should be in accordance with the relevant parameter that set in simulation), and its curve of the main cutting force of actual measurement is shown as Figure 6.

**Figure 7:**
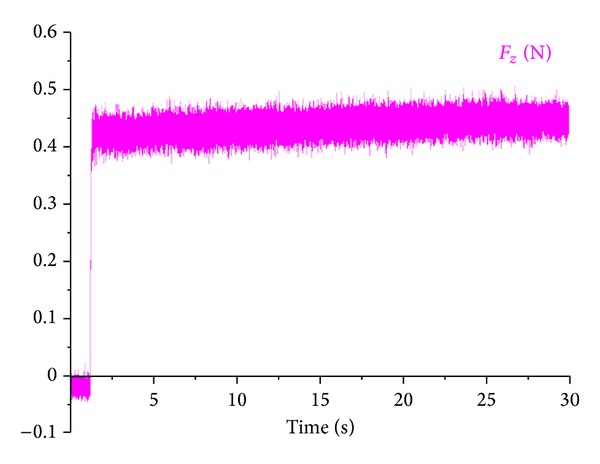
The main cutting force curve of dynamometer measurement.

In Figure 7, the trend of the main cutting force curve and the simulation curves is nearly identical; the actual cutting force is between 400 N and 500 N. Because there is great shake in actual work process, the fluctuation of the actual measured curve is greater than the simulation curve. Table 3 shows the average value of the actual measured cutting force under the condition of reaching a plateau which is compared to the average value of simulation; as shown in Table 5, the absolute error between average value of cutting force and the actual measured cutting force which are achieved by the means of finite element simulation is little; its predicted value well coincides with the experiment result, which meets the predicted requirement.

## 5. Conclusions

(1) In the case that the difference between cutting condition of experimental samples for modeling is not significant, the value of actual cutting force is put in the scope of fuzzy prediction by taking the membership grade *h*, which is ranging from 0.2 to 0.7, increasing the number of experimental samples so that the adaptability of the fuzzy prediction can be raised availably.

(2) Through comparing the predicted value which is achieved by the simulation of the thermomechanical coupled finite element and the actual measured value, it can be known that, in the scope of the experimental data, this method has a high prediction accuracy. And it can availably get the strain field and the temperature field, which cannot be measured directly in the cutting process of metal, simulating the whole turning process more intuitively.

(3) The study of the cutting force uncertainty using the integrated symmetric fuzzy number and finite element method has shown that, for the given cutting conditions, the predicted cutting force shows uncertain value which is fluctuated in a changeable scope instead a certain value based on the traditional method. It is important to realise the meaning for depth study to the metal cutting theory, predicting cutting force and optimizing the cutting parameters and so on.

(4) In the finite element numerical analysis, some important points such as establishing the constitutive relation have certain difference between the simulation result and the actual station.

## Figures and Tables

**Table 1:** The Johnson-Cook model characteristics parameters of AISI-1045 steel [7].

*A* (Mpa)	*B* (Mpa)	*n*	*m*	*T*_melt_ (°C)	*T*_Transition_ (°C)	*C*	${\dot{\overset{-}{\varepsilon }}}_{0}$ (s^−1^)
553	601	0.234	1.0	1460	20	0.0134	0.001

**Table 2:** The Johnson-Cook specified failure parameters of AISI-1045 steel [7].

*d*_1_	*d*_2_	*d*_3_	*d*_4_	*d*_5_
0.05	3.44	2.12	0.002	0.61

**Table 3:** The main cutting force measurement of AISI-1045 steel.

Serial number	Cutting speed (m/min)	Back engagement of cutting edge *a*_*p*_ (mm)	feed rate *f* (mm)	Cutting force *F* (N)
1	254	0.75	0.1	390.08
2	254	0.75	0.2	464.01
3	254	0.75	0.3	530.78
4	254	0.75	0.4	612.19
5	254	0.75	0.5	741.11
6	170	0.5	0.1	185.14
7	170	0.5	0.2	225.85
8	170	0.5	0.3	278.39
9	170	0.5	0.4	340.47
10	170	0.5	0.5	368.33
11	127	0.25	0.1	131.11
12	127	0.25	0.2	158.22
13	127	0.25	0.3	171.56
14	127	0.25	0.4	181.39

**Table 4:** The prediction of main cutting force by sculptured surface model (*h* = 0.5).

Serial number	Cutting speed (m/min)	Back engagement of cutting edge *a*_*p*_ (mm)	feed rate *f* (mm)	Measurement value (N)	predicted value (N)
1	254	0.75	0.1	390.08	[299.69, 468.72]
2	254	0.75	0.2	464.01	[366.04, 572.49]
3	254	0.75	0.3	530.78	[410.35, 641.80]
4	254	0.75	0.4	612.19	[445.80, 697.25]
5	254	0.75	0.5	741.11	[474.72, 742.48]
6	170	0.5	0.1	185.14	[174.58, 268.09]
7	170	0.5	0.2	225.85	[213.24, 327.44]
8	170	0.5	0.3	278.39	[239.04, 367.09]
9	170	0.5	0.4	340.47	[259.71, 398.80]
10	170	0.5	0.5	368.33	[276.55, 424.67]
11	127	0.25	0.1	131.11	[121.51, 131.63]
12	127	0.25	0.2	158.22	[148.41, 160.77]
13	127	0.25	0.3	171.56	[166.38, 180.24]
14	127	0.25	0.4	181.39	[179.72, 195.81]

**Table 5:** The simulation of force average values and the measured average values.

Serial number	Cutting speed (m/min)	Back engagement of cutting edge *a*_*p*_ (mm)	feed rate *f* (mm)	Measurement value (N)	Predicted value (N)	Absolute error (%)
1	254	0.75	0.1	390.08	369.44	5.29%
2	254	0.75	0.2	464.01	448.12	3.42%
3	254	0.75	0.3	530.78	508.75	4.15%
4	254	0.75	0.4	612.19	573.38	6.34%
5	254	0.75	0.5	741.11	698.27	5.78%
6	170	0.5	0.1	185.14	191.92	−3.66%
7	170	0.5	0.2	225.85	209.48	7.25%
8	170	0.5	0.3	278.39	290.86	−4.48%
9	170	0.5	0.4	340.47	359.3	−5.53%
10	170	0.5	0.5	368.33	336.91	8.53%
11	127	0.25	0.1	131.11	121.76	7.13%
12	127	0.25	0.2	158.22	169.11	−6.88%
13	127	0.25	0.3	171.56	183.59	−7.01%
14	127	0.25	0.4	181.39	166.81	8.04%
